# Nutritional and Chemical Characterization of Red and Purple Potatoes Peels: A Polyphenol-Rich By-Product

**DOI:** 10.3390/foods14101740

**Published:** 2025-05-14

**Authors:** Debora Dessì, Giacomo Fais, Giorgia Sarais

**Affiliations:** 1 Department of Biomedical Sciences, University of Sassari, 07100 Sassari, Italy; d.dessi14@phd.uniss.it; 2 Interdepartmental Center of Environmental Science and Engineering (CINSA), University of Cagliari, Via San Giorgio 12, 09124 Cagliari, Italy; giacomo.fais@unica.it; 3 Department of Mechanical, Chemical and Materials Engineering, University of Cagliari, Piazza d’Armi, 09123 Cagliari, Italy; 4 Department of Life and Environmental Sciences, University of Cagliari, 09123 Cagliari, Italy

**Keywords:** polyphenols, potato peels, anthocyanins, agri-food by-products, food industry sustainability

## Abstract

Potato peel represents a major by-product of the potato-processing industry and a promising source of bioactive compounds with potential health benefits. This study investigates the biochemical and nutritional composition of peels from five purple and two red potato cultivars, with particular attention to their phytochemical profiles and antioxidant properties. Total phenolic content, carbohydrates, proteins, and lipids were quantified using UV–visible spectrophotometry. The phytochemical composition was further characterized via High-Performance Liquid Chromatography coupled with a Diode-Array Detector (HPLC-DAD). Antioxidant and radical-scavenging capacities of the extracts were assessed through Ferric Reducing Antioxidant Power (FRAP) and 2,2-diphenyl-1-picrylhydrazyl (DPPH) assays. Significant variability was observed across cultivars for all measured parameters. While all samples were rich in carbohydrates and proteins, they shared a common phenolic profile dominated by chlorogenic acid and its derivatives, as well as caffeic acid. Anthocyanin composition, however, was highly cultivar-specific. Notably, all extracts demonstrated strong antioxidant and antiradical activities, in agreement with their high total phenolic content. These findings highlight the potential of red and purple potato peels as valuable sources of functional ingredients for food and nutraceutical applications.

## 1. Introduction

In the last 50 years, the production and accumulation of food by-products have increased. According to the Food and Agriculture Organization of the United Nations (FAO), agri-food processing industries generate huge quantities of by-products, estimated globally at 1.3 billion tons. These by-products cause pollution, and their disposal has demonstrated a negative impact on the environment and the economy of industries [1]. To overcome this problem, researchers have been adopting strategies based on a circular economic model to promote the reuse of by-products and obtain innovative products with high added value, thereby fostering sustainable bioeconomy growth among industries [2]. Potatoes (*Solanum tuberosum* L.) rank as the fourth most consumed food in the world [3], with a global production of around 370 million tons per year [4]. Potatoes are recognized for their high starch content, averaging 18% of fresh weight. They also contain substantial protein concentrations ranging from 1 to 1.5% of tuber fresh weight, while lipids represent the smallest fraction, amounting to 0.15% of fresh weight. Furthermore, raw potatoes are rich in essential minerals such as potassium, phosphorus, and calcium, which are integral to many enzymes and play a crucial role in metabolic regulation [5]. These micro- and macronutrients are distributed not only in the pulp but also in the peel, which therefore represents a good source of bioactive compounds, essential amino acids, vitamins, and minerals [6]. Less than 50% of the globally grown potatoes are consumed fresh, while the rest are processed into potato-based food products and ingredients by the industry. The peeling process in potato-processing industries generates significant quantities of by-products, with peel accounting for 6–10% of the total potato waste [7], equivalent to around 70–140 tons produced per year [8]. This results in a large amount of by-products containing a high content of value-added bioactive molecules, including polysaccharides, proteins, carbohydrates, vitamins, and polyphenolic compounds [9]. These compounds have demonstrated several beneficial properties for human health and exhibit potential for exploitation in the food, pharmaceutical, and nutraceutical industries [10,11]. Notably, phenolic acids, particularly chlorogenic acid (CGA), are widely accumulated in potato tuber peels and contribute to the high antioxidant activity of potatoes alongside hydroxycinnamic acids [12]. CGA has been reported to possess antidiabetic, anti-obesity, antioxidant, anti-inflammatory, antimicrobial, and anti-hypertension properties [13] and to slow down the entry of glucose into the bloodstream [5]. There are approximately 5000 cultivated potato varieties worldwide, with the main varieties featuring white–yellow flesh and skin [14]. Cultivated potato varieties exhibit considerable biodiversity, with a recent and increasing number of red- and purple-colored cultivars containing anthocyanins, a family of water-soluble bioactive flavonoids responsible for the vibrant colors such as red, blue, and purple observed in plants [15]. Interestingly, these phenolic compounds, which contribute to their unique chemical composition, are found in higher concentrations in the potato peel compared to the pulp [16]. Due to the high content of anthocyanins, these colored cultivars exhibit enhanced antioxidant, anticarcinogenic, anti-inflammatory, and neuroprotective activities and have demonstrated potential in preventing and treating diabetes and heart conditions [17]. Although some studies have reported on the nutritional and biochemical composition of purple potatoes, they have mainly focused on the pulp while neglecting the peel. Hence, in compliance with the European Commission’s circular economy action plan, the present study aims to investigate, for the first time, the biochemical and nutritional composition of the peels from five purple and two red potato cultivars and evaluate them as sources of healthy ingredients for the formulations of nutraceuticals.

## 2. Materials and Methods

### 2.1. Reagents and Standards

Concentrated sulfuric acid 96%, orthophosphoric acid 85%, sodium nitrate, potassium chloride, phenol, copper sulfate, sodium hydroxide, sodium potassium tartrate (all of RPE ACS Reagent Ph Eur grade), methanol (LC-MS grade), acetonitrile, calcium, iron, phosphorus, magnesium, manganese, potassium, sodium, zinc, aluminum, boron, barium, chromium, copper, strontium, vanadium, and nickel (all of RS standard solution for ICP) were purchased from Carlo Erba (Val de Reuil Cedex, France). Chloroform (RPE ACS Reagent Ph Eur grade), hydrogen peroxide solution 30% (for analysis), sodium carbonate, Folin–Ciocalteau reagent, analytical standards of gallic acid, caffeic acid, chlorogenic acid, malvidin-3-glucoside albumin, and vanillin were purchased from Sigma-Aldrich (Merck, Milan, Italy). 2,2-diphenyl-1-picrylhydrazyl (DPPH), 2,4,6-tris(2-pyridyl)-s-triazine (TPTZ), ferric chloride, 6-hydroxy-2,5,7,8-tetramethylchromane-2-carboxylic acid (Trolox), and ferrous sulfate were purchased from Sigma-Aldrich (St. Louis, MO, USA). For standard lipid stock, commercial canola oil was obtained from a local market. For aqua regia preparation, superpure nitric acid 67–69%, and superpure chloridric acid 34–37% (for trace analysis, Carlo Erba, Milan, Italy) were used. Ultrapure water (conductivity lower than 18.2 MΩ) was distilled and filtered through a Milli-Q system (Millipore, Bedford, MA, USA).

### 2.2. Samples

This study focused on five purple potato cultivars: Patate e’moru, Violet queen, Fleur bleue, Blue star, Blawe borges, and two stunning red varieties, Magenta love, and Rote emma. These potato samples were generously provided by Radici d’Ogliastra, a local agricultural company based on the east–central coast of Sardinia (Italy).

To prepare for chemical analysis, the potato peels, delicately shaved to a thickness of ~1 mm using a household potato peeler, were flash-frozen in liquid nitrogen to preserve their integrity. They were then carefully lyophilized using a freeze dryer (LIO 5P DGT, Cinquepascal, Trezzano s/Naviglio, MI, Italy). Once dried, the samples were finely ground in an electric coffee grinder, transforming them into a uniform, silky powder. These powders were stored in a cool, dark environment with low humidity until ready for extraction and analysis, ensuring optimal preservation of their properties.

### 2.3. Macronutrients Determination

#### 2.3.1. Total Carbohydrate Determination

The total carbohydrate content was quantified using a colorimetric method, slightly adapted from the protocol described by Dubois et al. [18]. To begin, 10 mg of freeze-dried, finely ground sample was reconstituted in 10 mL of distilled water. The mixture was subjected to ultrasonic treatment for 30 min in an ultrasonic bath (Ultracleaner 040S, Be-Right (Medical) Co., Ltd., Foshan, China) to enhance extraction efficiency. An aliquot of the resulting sample or a glucose standard solution was combined with 200 µL of 5% (*w*/*v*) phenol and 1 mL of concentrated sulfuric acid. The reaction mixture was incubated at room temperature for 30 min for color development. Absorbance was then measured at 490 nm against a blank sample using a Varian Cary 50 UV–Vis Spectrophotometer (Agilent Technologies, Woodburn, Australia) and standard 1 cm disposable cuvettes. Quantification was performed via an external calibration curve generated with glucose as the reference standard. Each analysis was conducted in triplicate to ensure reproducibility, with results expressed as g/100 g dry weight (DW) ± standard deviation (SD) of glucose.

#### 2.3.2. Determination of Total Protein

To determine the protein content, the analysis was carried out according to the method of Lowry et al. [19], appropriately modified. For this analysis, the same extract prepared for carbohydrate quantification was utilized. In total, 500 µL of the extract or standard (albumin) was added to 500 µL of sodium hydroxide 1N and heated at 100 °C for 5 min to facilitate protein solubilization.

Subsequently, 2.5 mL of a reagent containing 5% (*w*/*v*) sodium carbonate, 0.5% (*w*/*v*) copper sulfate, and 1% (*w*/*v*) sodium potassium tartrate was added to the mixture. After a 10 min reaction period, 500 µL of 1N Folin–Ciocalteu reagent was introduced. The mixture was incubated at room temperature for 30 min to enable the formation of the colored complex. The absorbance of the samples was measured at 750 nm against a reagent-only blank using a UV–Vis spectrophotometer (Varian Cary^®^ 50). Protein quantification was performed using an external calibration curve prepared with albumin as the standard. All measurements were carried out in triplicate, with the protein content expressed as g/100 g DW ± SD of albumin.

#### 2.3.3. Total Lipids Determination

Lipids determination follows lipids extraction carried out according to the methods by Chen et al. [20] and Bligh e Dyer [21]. In total, 100 µL of PBS and 1.5 mL of sodium hydroxide 1 N containing 25% of methanol were added to the lyophilized samples (15 mg). The suspension was shaken with a vertical rotary mixer Falc F200 (Falc Instruments s.r.l, Treviglio (BG), Italy) and heated at 100 °C. The suspension was centrifuged at 4000 rpm for 15 min (Centrifuge 5810 R, Eppendorf, Milan, Italy); then, 1 mL of supernatant was withdrawn and added to a methanol/chloroform solution 1:2 (*v*/*v*) and 0.5 mL of potassium chloride solution 0.88% (*w*/*v*). The suspension was shaken with a vertical mixer F200 (Falc instruments s.r.l, Treviglio (BG)) and centrifuged at 4000 rpm for 15 min.

To determine total lipids content, a colorimetric test of the chloroformic supernatant was conducted according to the protocol of Mishra et al. [22], slightly modified.

Briefly, 1 mL of chloroform phase was put into a glass vial, evaporated to dryness under a gentle stream of nitrogen, and 100 µL of concentrated sulfuric acid was added. After keeping the vial in the oven at 90 °C for 10 min, 2.4 mL of 68% (*w*/*v*) phosphovanillin reagent was added to the sample. After a further 10 min of incubation at room temperature, the OD was measured against a blank at 530 nm using 1 cm wide disposable cuvettes.

A quantitative analysis was carried out using an external standard calibration method using oil containing 100% fat as a reference. All the analyses were conducted in triplicate, and the results were expressed in g/100 g DW ± SD of canola oil.

### 2.4. Micronutrients Determination

The concentrations of metals such as calcium, iron, phosphorus, magnesium, manganese, potassium, sodium, zinc, aluminum, boron, barium, chromium, copper, strontium, vanadium, and nickel were determined using an Agilent 5100 optical emission spectrometer (ICP-OES) following acid digestion. Specifically, 0.2 g of sample was mineralized using a CEM Mars6 microwave system (CEM Corporation, Milan, Italy) by adding 3 mL of aqua regia and 1 mL of H_2_O_2_. The microwave digestion process was conducted with the following operational parameters: power set at 600 W for 3 min at 50%, 600 W for 2 min at 60%, 600 W for 3 min at 70%, and 600 W for 10 min at 80%. After cooling, the digested solution was transferred to a 25 mL volumetric flask, diluted to volume with Milli-Q water, and filtered through a 0.45 µm nitrocellulose membrane filter (Corning S.P.A, Pisa, Italy). Quantification of each metal was achieved using the external standard method, correlating the light intensity at the selected wavelengths with the concentrations of reference standards. The analytical wavelengths (nm) used for each metal were as follows: calcium (422.673), iron (238.204), phosphorus (213.618), magnesium (279.553), manganese (257.610), potassium (766.491), sodium (589.592), zinc (202.548), aluminum (237.312), boron (249.772), barium (455.403), chromium (267.716), copper (213.598) strontium (407.771), vanadium (311.837), and nickel (230.299).

### 2.5. Polyphenols Extraction

Polyphenol extraction was carried out following the procedure outlined by D’Amelia et al. [23]. According to this method, in this process, a sample aliquot was subjected to extraction using an ultrasonic cleaner (040S model, AC220-240V 50 Hz, heating power 200 W, frequency 40 KHz, time 0–30 min, Be-Right Medical) Co., Ltd., Foshan, China) for 1 h, utilizing a 70% ethanol solution in water. The resulting suspension was then centrifuged with an Eppendorf Centrifuge 5810/5810 R (Eppendorf, Milan, Italy) at 4000 rpm for 30 min at a controlled temperature of 10 °C. Following this, the extracts were filtered through a 0.45 µm nylon filter (Corning S.P.A, Pisa, Italy) and injected directly into the HPLC system after dilution with 0.22 M orthophosphoric acid.

### 2.6. HPLC Polyphenols Analysis

An Agilent HPLC 1100 liquid chromatograph coupled with a Thermo Finnigan DAD CHROMQUEST UV 6000 diode array detector was used to perform the analysis. Chromatographic separation was obtained according to D’Amelia et al. [23]. The column was a Kinetex 5 µm (C18 100A—150 × 4.6 mm, Phenomenex). A 10 µL volume sample was injected and eluted in 120 min (0.4 mL/min) via a binary gradient mobile phase consisting of H3PO4 0.22 M (solvent A) and Acetonitrile (solvent B) with the following gradient elutions: 0 min, 95% A—5% B; 30 min 90% A–10% B; 35 min, 85% A–15% B; 70 min, 70% A–30% B; 100 min, 10% A–90% B; 120 min, 100% B.

At the end of each run, the column was reconditioned for 15 min. Considering the chemical structure of main phenolic compounds, two distinct wavelengths were used: 280 nm and 520 nm for the determination of chlorogenic acid, caffeic acid and its derivatives, and anthocyanins, respectively. Peaks’ identification was carried out by comparing each compound’s retention time and UV spectra with the reference standard. The identification of anthocyanins was performed by comparing their retention times and UV spectra with data reported in the literature. Individual phenolic compounds were quantified using an external standard calibration method. Correlation values ranged between 0.9990 and 0.9999. The calibration curve was prepared in orthophosphoric acid 0.22 M from a suitable dilution of the standard stock solution (1000 mg L^−1^ in methanol).

### 2.7. Determination of Total Polyphenols

The Folin–Ciocalteu assay, a reference method for quantifying total polyphenols, was employed with a modified protocol adapted from Singleton, V. L. (1965) [24]. Briefly, 100 µL of the extract or gallic acid standard was added to 500 µL of the Folin–Ciocalteu reagent and allowed to react for 5 min at room temperature. Afterwards, 3 mL of a 10% (*w*/*v*) sodium carbonate (Na_2_CO_3_) solution were added, and ultrapure water was added to reach a final volume of 10 mL. After incubation for 90 min at room temperature, the optical density (OD) was recorded at 725 nm using a Varian Cary 50 spectrophotometer and disposable cuvettes with a 1 cm path length. Quantitative analysis was performed using an external calibration curve, and the results were expressed as mg/g of gallic acid equivalent (GAE).

### 2.8. DPPH Radical-Scavenging Assay

The modified method proposed by Brand-Williams et al. [25] was used for the DPPH (1,1-diphenyl-2-picryl-hydrazyl) assay. A total of 20 µL of the extract or standard (Trolox) was added to 2 mL of a 40 µM methanolic DPPH solution. After a 90 min incubation at room temperature, the optical density (OD) was measured at 517 nm against a blank using 1 cm wide disposable cuvettes. Quantitative analysis was conducted using an external standard calibration method, and the results were expressed as mmol/g of TEAC (Trolox equivalent antioxidant capacity).

### 2.9. FRAP: Ferric-Reducing Antioxidant Power

The FRAP (Ferric-Reducing Antioxidant Power) assay was achieved following a slightly modified protocol based on Axelrod et al. [26]. The reagent was prepared by mixing TPTZ (10 mM) and ferric chloride (20 mM) in acetate buffer (pH 3,6). In total, 50 μL of diluted extract 1:10 (*v*/*v*) or standard (ferrous sulfate) was added to 2 mL of this solution. After 4 min of incubation at room temperature, the OD was measured against a blank at 593 nm using 1 cm wide disposable cuvettes. Quantitative analysis was carried out using an external standard calibration method, and the results were expressed in mmol/g of reduced iron of ferrous sulfate.

### 2.10. Statistical Analysis

The datasets were imported into the PLS_Toolbox (version 7.5.1, Eigenvector Research, Manson, WA, USA) where the parameters were analyzed by principal component analysis (PCA) and ANOVA. Statistical analyses were carried out using GraphPad PRISM 8.00 (GraphPad Software, San Diego, CA, USA). Data were expressed as mean ± SD (standard deviation). For data analysis, one-way ANOVA followed by Tukey’s multiple comparisons post hoc test. Means ± SD denoted by the same letter did not differ significantly at *p* ≤ 0.05, while different letters denote statistical differences with at least 95% confidence according to Tukey’s multiple comparisons test.

## 3. Results and Discussion

### 3.1. Nutritional Composition

#### 3.1.1. Macronutrients

Recently, consumer demand for food products rich in antioxidants and enhanced with high levels of fiber and protein has increased, thanks to their acknowledged health benefits [27]. Potatoes are a staple in the human diet, providing vital biologically active compounds such as starch, fiber, minerals, and polyphenols. The presence of anthocyanins in colored potatoes has further boosted their popularity.

However, potato peeling and processing produce waste materials that retain their nutritional properties and biological activity, containing significant levels of valuable substances like polyphenols, starch, proteins, and fibers [9,28,29,30,31].

In this study, we analyzed the waste generated from peeling seven colored potato varieties, focusing on the primary nutrients and key active components in the functional fraction of the potatoes.

Beginning with the analysis of macronutrients, the findings demonstrate notable compositional differences between nutrients and across various parameters among the cultivars, as shown in Table 1. The grouping of lipids, determined through one-way ANOVA and subsequent Tukey’s multiple comparisons test—reflected by superscript letters in the lipid content figures—supports these observations.

Potatoes are not deemed a significant source of lipids; therefore, it is expected that their peels have a low lipid content, which our research has validated. The lipid values hovered around 1%, with a noted range from 0.7 ± 0.0 g/100 g DW in Fleur bleue to a peak of 1.3 ± 0.1 g/100 g DW in Magenta love. A significant difference (* *p* < 0.05) was noted between Patata e’moru (0.9 ± 0.0 g/100 g DW) and Magenta love, with the latter showing a distinctly higher lipid content. Likewise, Fleur bleue, which had the lowest lipid content, was significantly (* *p* < 0.05) lower than Violet queen (1.0 ± 0.1 g/100 g DW), Blawe borges (1.2 ± 0.1 g/100 g DW), and Magenta love. Blue star (0.8 ± 0.1 g/100 g DW) also demonstrated a significant difference when compared to Blawe borges (* *p* < 0.05) and Magenta love (** *p* < 0.01), with the latter two varieties showing greater lipid content. Furthermore, the comparison between Blawe borges and Rote emma (0.8 ± 0.1 g/100 g DW) confirmed that Blawe borges had a significantly (* *p* < 0.05) higher lipid content than Rote emma. Likewise, Magenta love exhibited a significantly higher (** *p* < 0.01) lipid content compared to Rote emma. Varieties including Patata e’moru, Violet queen, Blue star, and Rote emma exhibit similar letters, suggesting comparable lipid content. In contrast to lipids, proteins are vital to the nutritional profile of potatoes. They play a crucial role in human diet, acting as significant dietary supplements [32]. Potatoes are, in fact, a rich source of high-quality proteins and essential amino acids, particularly lysine [33].

Colored potatoes contain between 4.4 and 12.5 g/100 g of protein dry weight (DW), comparable to traditional yellow-fleshed potatoes, which have a range of 5 to 13 g/100 g DW, making them valuable food sources [34]. A comparative study of plant and animal proteins shows that potato protein has a higher biological value than many crops and is on par with animal proteins, such as casein, noted for being highly digestible [35]. This indicates that potato proteins could potentially supply adequate essential amino acids, promoting effective muscle protein synthesis [36].

Surprisingly, despite the common perception of potatoes being associated mainly with high carbohydrate content, an analysis of macronutrients reveals that potato peels can have notably high protein levels, although there can be substantial variation (up to 2.5-fold) among different varieties in protein concentration, reflecting the nutritional diversity of each cultivar.

The Violet queen variety showed the highest protein content (31.8 ± 3.0 g/100 g DW), significantly surpassing all other cultivars (**** *p* < 0.0001) except Magenta love, which also had a high protein level (27.9 ± 2.0 g/100 g DW), both ranking among the highest nutritional value varieties. This could imply either superior protein accumulation capacity or a unique genetic makeup that enhances protein synthesis. The protein content of Patata e’moru (24.4 ± 2.6 g/100 g DW) was statistically comparable to that of Magenta love but significantly lower (* *p* < 0.05) than Violet queen. Rote emma recorded an intermediate protein content (19.5 ± 0.3 g/100 g DW), which was higher than Blue star and Blaw borges but lower than Violet queen and Magenta love. Lastly, Fleur bleue (13.9 ± 3.2 g/100 g DW), Blue star (12.1 ± 0.9 g/100 g DW), and Blaw borges (12.8 ± 0.5 g/100 g DW) had significantly lower protein contents compared to the aforementioned cultivars. These values indicate a lower protein concentration relative to the higher-performing varieties, suggesting possible differences in metabolic processes governing protein accumulation. Generally, the lower protein levels observed are consistent with previous studies where most potato peel varieties contained between 2 and 16 g/100 g DW of total protein matter [37,38]. Beyond the protein content, the carbohydrate composition of potato peels is also crucial in defining their overall nutritional value. Increasing attention is directed toward certain types of carbohydrates that, although indigestible by human enzymes, are vital for physiological functions through their interactions with the gut microbiota [39,40]. Consequently, a fiber-rich diet offers numerous health benefits, including a decreased risk of cardiovascular disease [41], reduced cholesterol levels, and a lower risk of atherosclerosis [42]. Furthermore, it is noted for its preventive effects against certain cancers like colorectal cancer [43], as well as conditions like metabolic syndrome [44], inflammatory bowel syndrome [45], diverticular disease [46], and diabetes [47]. Recently, innovative fiber sources have presented economically viable options for developing new functional foods. One such source is the by-product fraction obtained from various food-processing methods involving fruits and vegetables, including potato peels [48,49].

In this study, various potato varieties were analyzed to compare their carbohydrate content and evaluate significant differences. As anticipated, carbohydrates constitute the most abundant nutrient, comprising at least 70% of the total macronutrient content. The data indicate that the carbohydrate content in the studied potatoes ranges from 77.5 ± 3.0 g/100 g DW for Blue star to 95.1 ± 4.2 g/100 g DW for Fleur bleue. Other varieties, including Patata moru (87.4 ± 3.0 g/100 g DW), Violet queen (78.7 ± 7.9 g/100 g DW), Blaw borges (89.7 ± 7.4 g/100 g DW), Magenta love (82.0 ± 8.0 g/100 g DW), and Rote emma (92.6 ± 1.2 g/100 g DW), fall within an intermediate range. This variability may arise from factors such as cultivation conditions and soil composition. Despite the numerical differences, they were not statistically significant, suggesting that, from a nutritional perspective, all analyzed varieties offer a comparable carbohydrate content.

#### 3.1.2. Mineral Content

The mineral distribution within plant structures differs based on the specific component examined, such as the contrast between potato skins and pulp. Notably, from a nutritional standpoint, even potato skins, often seen as waste, serve as a rich source of minerals, particularly potassium, sometimes exceeding the mineral levels found in the pulp, as Wszelaki emphasizes [50]. However, despite numerous studies on the mineral makeup of yellow potato varieties, knowledge regarding the mineral profiles of colored potato skins is scarce and generally pertains to specific cultivar groups [37,38].

Moreover, it is essential to acknowledge that variations in mineral content stem not only from potato genotype but also from agricultural practices and environmental conditions, as highlighted by Nijolė Vaitkevičien [38]. Focusing on purple potato skins, research by Nijolė Vaitkevičien found four macroelements (K, P, Ca, and Mg) alongside five microelements (Fe, Zn, B, Mn, and Cu) across various colored potato cultivars. Similarly, a study conducted by Bellumori et al. identified five macroelements (K, P, Ca, Na, and Mg) and four microelements (Fe, Zn, Mn, and Cu) within the skins of these cultivars. Consistent with these observations, our investigation identified and quantified the primary macroelements (K, Ca, Mg, Na, and P) as well as three crucial microelements (Fe, Mn, and Zn), detailed in Table 2. Additionally, we examined other trace elements such as Al, B, Ba, Cr, Cu, Sr, V, and Ni. Nevertheless, the concentrations of these supplementary elements remained under 1 mg/100 g of dry weight across all potato varieties. Given their extremely low levels and minimal variations among cultivars, they were deemed irrelevant for the comparative nutritional assessment presented in this study. Indeed, the main focus was placed on elements that exhibited quantifiable concentrations surpassing the nutritional significance threshold and showcased marked inter-varietal differences.

Our analysis revealed notable variations among all the investigated cultivars, as outlined in Table 2, with potassium (K) standing out as the dominant mineral element in all cultivars, corroborating findings from Nijolė Vaitkevičien and Bellumori [37,38]. It is important to highlight the significant differences in the less common mineral elements across various cultivars.

The evaluation of various potato varieties showed notable differences in K values, demonstrating that certain types are clearly superior. Notably, the Violet queen achieved the highest K value at 2375.4 ± 9.4 mg/100 g DW, significantly surpassing Patata e’moru (1399.6 ± 22.4 mg/100 g DW) (**** *p* < 0.0001), Fleur bleue (1475.4 ± 179.6 mg/100 g DW) (**** *p* < 0.0001), and Rote emma (1649.1 ± 9.7 mg/100 g DW) (**** *p* < 0.0001). In contrast, Patata e’moru had one of the lowest readings, as previously noted, being significantly less than other varieties analyzed, including Blue star (2135.8 ± 243.6 mg/100 g DW) (**** *p* < 0.0001) and Magenta love (2130.0 ± 24.4 mg/100 g DW) (**** *p* < 0.0001). The Blawe borges variety (1893.4 ± 21.8 mg/100 g DW) showed intermediate results, falling between the K values of Patata e’moru and Violet queen. Earlier research by Nijolė Vaitkevičien and Bellumori et al. indicated potassium concentrations ranging from 2430 to 3330 mg/100 g DW and from 1241 ± 209.6 to 1738.6 ± 211.1 mg/100 g DW, respectively. Considering the suggested daily intake of 2500 mg/day, potato peels are regarded as a valuable source of dietary potassium, serving as a supplement during shortages caused by intense heat or physical exertion [51].

Calcium ranks as the second most abundant element and displays considerable variability among the cultivars analyzed. The varieties Patata e’moru, Violet queen, and Magenta love exhibited the highest and most statistically comparable calcium concentrations, measuring 621.3 ± 20.2, 663.2 ± 35.7, and 602.5 ± 109.4 mg/100 g DW, respectively. Rote emma also presented a notable calcium content of 524.5 ± 11.4 mg/100 g DW, albeit with some statistical differences relative to other cultivars. The cultivars with the least calcium content were Fleur bleue (415.1 ± 6.4 mg/100 g DW) and Blawe borges (370.2 ± 3.3 mg/100 g DW), which were statistically similar to Blue star (488.5 ± 22.0 mg/100 g DW) and significantly lower than Patata e’moru, Violet queen, and Magenta love.

Our study revealed calcium concentrations that were ten times greater than those documented in other research [37,38]. This notable discrepancy is likely associated with environmental factors. Initially, soil type can significantly impact calcium availability, as soils enriched with minerals or possessing certain pH levels can enhance calcium uptake by plants [52]. Additionally, the distribution of nutrients, often shaped by local agricultural practices, fertilization histories, or geographical proximity to calcareous materials, can result in concentrated nutrient levels [53]. Furthermore, factors like sampling depth and timing, which can differ between studies, may greatly influence the observed calcium levels, since nutrient concentrations are not consistently uniform across the soil profile or through seasonal variations [54].

Magnesium is a vital mineral frequently missing from diets. The recommended daily intake is between 250 and 350 mg/day [55]. Our sample magnesium levels range from 141.4 ± 4.5 mg/100 g DW in Patata e’moru to 184.5 ± 12.7 mg/100 g DW in Magenta love. Statistical analysis reveals two distinct groups for magnesium levels across various potato types. The first group, including Magenta love, Violet queen (179.2 ± 4.5 mg/100 g DW), and Rote emma (178.8 ± 2.8 mg/100 g DW), shows significantly elevated Mg levels compared to the second group, which consists of Patata e’moru, Fleur bleue (154.5 ± 11.7 mg/100 g DW), Blue star (148.9 ± 14.0 mg/100 g DW), and Blawe borges (153.4 ± 4.0 mg/100 g DW). The magnesium content in purple potato peel corresponds with the findings of Nijolė Vaitkevičien (ranging from 119 to 160 mg/100 g DW), while research by Bellumori et al. indicated lower magnesium levels (41.4 to 83.3 mg/100 g DW) [37,38].

Sodium concentrations varied widely across cultivars. Rote emma (93.0 ± 1.4 mg/100 g DW) and Violet queen (89.9 ± 7.1 mg/100 g DW) had the highest sodium content, followed by Patata e’ Moru (68.6 ± 5.8 mg/100 g DW), which showed significant differences compared to Rote emma and Violet queen. Magenta love exhibited a significantly lower sodium level (** *p* < 0.01) at 49.0 ± 4.3 mg/100 g DW compared to Patata e’ Moru. In contrast, Fleur bleue, Blu Star, and Blawe borges presented the lowest sodium concentrations (23.5 ± 2.9, 23.6 ± 2.3, and 25.6 ± 0.9 mg/100 g DW, respectively), with no significant differences among them.

Phosphorus, an essential element for tuber growth and overall bodily functions, showed varying levels among cultivars, ranging from 13.1 ± 0.1 to 33.4 ± 3.2 mg/100 g DW. The comparative analysis of the potato varieties listed in the table revealed significant differences among the various groups regarding the mean values obtained, illustrating diverse responses among the varieties. Notably, the Blue star variety (33.4 ± 3.2 mg/100 g DW) emerged as the highest, with mean values considerably surpassing those of all the other varieties analyzed, distinguishing it clearly from the rest. This notable difference may suggest a superior yield or enhanced performance in specific parameters for the Blue star variety, potentially related to traits, such as resilience to environmental stress or adaptability to various cultivation conditions.

In comparison, the Patata e’moru (14.2 ± 0.4 mg/100 g DW), Fleur bleue (14.7 ± 1.6 mg/100 g DW), and Blawe borges (13.1 ± 0.1 mg/100 g DW) varieties showed significantly lower values than the others. Intermediate values were noted for the Violet queen (20.6 ± 0.1 mg/100 g DW), Magenta love (20.0 ± 0.4 mg/100 g DW), and Rote emma (19.2 ± 0.4 mg/100 g DW) varieties, indicating no significant differences among these varieties, as illustrated by the statistics in Table 2. Unlike calcium concentrations, these values were ten times lower than those found in other studies [37,38].

Fe content displayed considerable variation across different cultivars. Rote emma had the highest concentration, measuring 71.3 ± 6.1 mg/100 g DW, significantly outpacing all other cultivars. A notably high Fe content was also present in Violet queen (51.4 ± 2.2 mg/100 g DW), which was statistically distinct from the other groups.

An intermediate Fe concentration was observed in Magenta love (29.7 ± 1.1 mg/100 g DW), overlapping statistically with Blue star (21.9 ± 2.7 mg/100 g DW), suggesting their Fe contents may not differ significantly. Lastly, the lowest Fe concentrations were recorded in Patata e’Moru (18.7 ± 1.7 mg/100 g DW), Fleur bleue (12.6 ± 1.3 mg/100 g DW), and Blawe borges (17.2 ± 1.2 mg/100 g DW), which were statistically similar to one another. Moreover, these cultivars showed no significant differences from Blue star, indicating a relatively uniform Fe content among these samples. Among the analyzed micro-elements (Fe, Zn, and Mn), iron was present in the highest concentration. Iron and zinc deficiencies are the most common forms of micronutrient malnutrition, presenting serious health risks [56].

Manganese and zinc had the lowest concentrations among the minerals assessed. Specifically, the Fleur bleue variety (1.6 ± 0.0 mg/100 g DW) showed the lowest zinc levels compared to other varieties. Notably, a comparison between Fleur bleue and Patata e’ moru (3.3 ± 0.3 mg/100 g DW) reveals a significant difference (* *p* < 0.05), with Patata e’ moru having higher values. This suggests that both varieties rank among the highest in this study. The other varieties, including Blue star (2.8 ± 0.7 mg/100 g DW), Blawe borges (2.8 ± 0.2 mg/100 g DW), Magenta love (3.1 ± 0.4 mg/100 g DW), Violet queen (3.7 ± 0.2 mg/100 g DW), and Rote emma (3.0 ± 0.4 mg/100 g DW), have values that do not show statistically significant differences in most cases.

When it comes to manganese, Rote emma emerges as the clear leader with a concentration (2.6 ± 0.0 mg/100 g DW), significantly higher than all other varieties. This notable difference emphasizes its exceptional performance in the measured aspects, distinguishing it from the other analyzed varieties. The significant manganese content in Rote emma is nutritionally important, given manganese’s role as an essential trace element that aids in bone formation, metabolism, and antioxidant functions [57]. This amount is meaningful for dietary intake, as the recommended daily intake for adults is around 3 mg [58]. Consequently, Rote emma may serve as a valuable manganese source in a balanced diet.

In the mid-range, Violet queen (2.1 ± 0.0 mg/100 g DW) and Magenta love (1.8 ± 0.2 mg/100 g DW) show comparable values, with no statistically significant differences between them.

Conversely, the lower-tier varieties—Patata e’ moru (1.3 ± 0.0 mg/100 g DW), Fleur bleue (1.2 ± 0.1 mg/100 g DW), Blue star (1.3 ± 0.1 mg/100 g DW), and Blawe borges (1.2 ± 0.1 mg/100 g DW)—display significantly lower values.

In summary, the analysis reveals a distinct gap between Fleur bleue, which has the lowest values, and Patata e’ moru and Violet queen, which rank as the varieties with the highest values.

The remaining varieties fall within an intermediate range without significant differences among them.

### 3.2. Functional Composition

#### 3.2.1. Total Polyphenol Content and Antioxidant Potential

**Figure 1:**
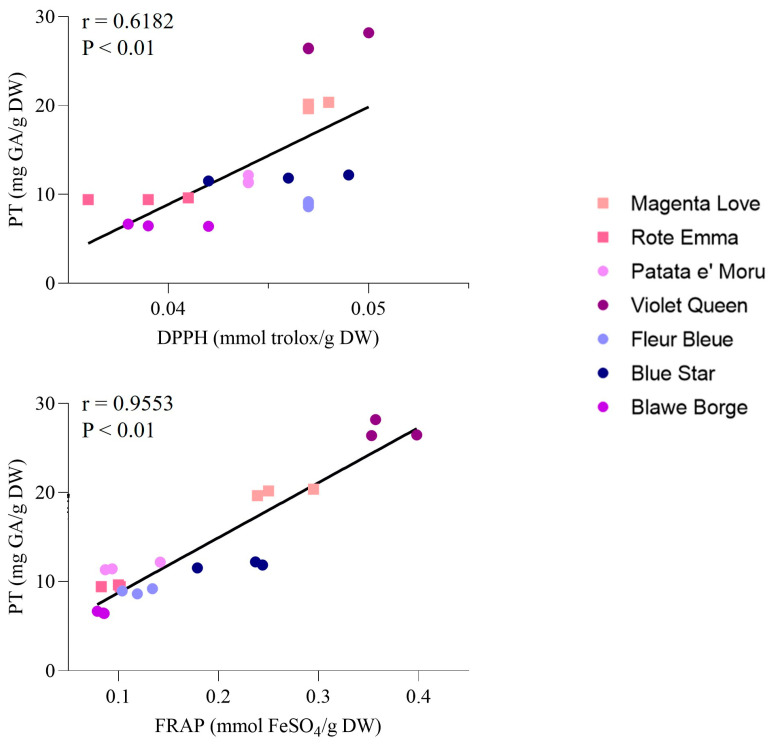
Correlation analysis between total polyphenols content (TP), antioxidant activity (DPPH) and antiradical activity (FRAP).

It is well-known that diets high in polyphenols are beneficial for the human body, largely due to their antioxidant properties [59]. Colored potatoes have therefore been researched for their antioxidant potential, and it has been extensively shown that their extracts can yield positive biological effects. Research by Shiyu Li et al. demonstrated that purple-fleshed potatoes have anti-colitic effects through modulation of the gut microbiome, as well as affecting oxidative stress and inflammation in a well-established acute murine colitis model [60]. In vitro tests have also shown these extracts possess antitumor properties linked to their capacity to induce apoptosis via ROS production [61,62]. Additionally, their potential antimicrobial effects [63] and their ability to lower blood cholesterol levels induced by a high-fat diet are noteworthy [64]. The biological effects of purple potato peel extracts remain relatively unexplored, with only a handful of scientific publications addressing this area [65,66,67,68,69]. This research indicates that the beneficial effects of peel extracts stem from phytochemicals, which tend to be present in higher amounts in the peels compared to the flesh [16]. Analyzing their composition and antioxidant capabilities can provide important insights for the valorization of agro-food waste. Specifically, assessing total polyphenol (TP) content, DPPH radical-scavenging activity, and ferric-reducing antioxidant power (FRAP) values in purple and red potato tubers provide valuable information regarding the antioxidant potential of various cultivars. As indicated in Table 3, the findings reveal significant differences among cultivars concerning TP content, DPPH radical-scavenging activity, and FRAP values. For instance, the Violet queen cultivar showed the highest TP content (27.00 ± 1.02 mg/g DW), showcasing its rich phenolic profile. Magenta love also exhibited substantial TP content (20.06 ± 0.37 mg/g DW). Other notable TP concentrations were found in Fleur bleue (8.91 ± 0.29 mg/g DW) and Blue star (11.85 ± 0.34 mg/g DW), while Blawe borges had the lowest TP content (6.51 ± 0.13 mg/g DW). In terms of DPPH radical-scavenging activity, Violet queen recorded the highest level (0.048 ± 0.002 mmol/g DW), closely followed by Fleur bleue (0.047 ± 0.000 mmol/g DW) and Magenta love (0.047 ± 0.000 mmol/g DW), indicating relatively strong antioxidant activity among these varieties. In contrast, Rote emma and Blawe borges showed the lowest DPPH radical-scavenging activities at 0.039 ± 0.003 mmol/g DW and 0.040 ± 0.002 mmol/g DW, respectively. The FRAP values identified Violet queen as the top performer (0.369 ± 0.030 mmol/g DW), highlighting its strong antioxidant potential. Magenta love (0.261 ± 0.030 mmol/g DW) and Blue star (0.240 ± 0.005 mmol/g DW) also presented significant FRAP values, while Blawe borges had the lowest FRAP value (0.083 ± 0.004 mmol/g DW). Overall, these findings underline significant differences in polyphenol content and antioxidant activity among the purple and red potato cultivars, with Violet queen and Magenta love emerging as promising options with high TP content and strong antioxidant properties. Furthermore, a correlation between TP and antioxidant activity has been established, with Pearson’s correlation coefficients presented in Figure 1. A moderate positive correlation was found between TP and DPPH (r = 0.6182, *p* < 0.01), along with a strong correlation between TP and FRAP (r = 0.9553, *p* < 0.01). Our findings align with those reported by Liqin et al., though analyses were performed on different genotypes of colored potatoes, where any discrepancies may arise from variations in extraction and analytical methods [70]. Albishi et al. also demonstrated that the peel of purple potatoes had higher antioxidant activity associated with a strong total phenolic content, indicating that anthocyanins and phenolic compounds were primarily responsible for the antioxidant capacity in the colored potato peel. Furthermore, when comparing the data on the antioxidant activity of purple potato peel and yellow potato peel, it can be concluded that the presence of anthocyanins makes a greater contribution to antioxidant activity [71].

#### 3.2.2. Quantitative Analysis of Polyphenols in Red and Purple Potatoes Peel

In nutrition, polyphenols are increasingly recognized by nutritionists, researchers, and experts for their biological and pharmacological benefits [72]. An analysis of total polyphenols in plant-based foods has shown that by-products contain significant amounts of valuable phenolics, making them a reference point for supplement production. Notably, potato peels, a by-product of the potato industry, have higher levels of polyphenols than the flesh itself. Gebrechristos et al. identified the most prevalent polyphenols in the peel, ordered by decreasing concentration: caffeic acid, chlorogenic acid, and neochlorogenic acid [73]. Other research also emphasizes chlorogenic acid and caffeic acid as the primary phenolic compounds in potato peels, with notable differences in chlorogenic acid levels across various potato varieties. Furthermore, pigmented cultivars generally have higher concentrations of chlorogenic acid and caffeic acid compared to their non-pigmented counterparts [74,75]. The existing literature also notes the presence of phenols in lower quantities, including caffeoylquinic and feruoylquinic derivatives, along with a range of phenolic acids, such as gallic acid, protocatechuic acid, vanillic acid, p-hydroxybenzoic acid, and p-coumaric acid [76,77,78]. As noted by Visvanathan, chlorogenic acid (CGA) is the primary phenolic acid in potatoes, making up more than 90% of the total phenolic content [75]. Our results align with this finding and show considerable variation in CGA levels across the potato cultivars analyzed (Table 4), with concentrations ranging from 1.18 ± 0.04 to 10.53 ± 0.95 mg/g DW. Notably, Violet queen had the highest CGA concentration at 10.53 ± 0.95 mg/g DW, followed by Blue star (6.45 ± 0.28 mg/g DW), Rote emma (5.06 ± 0.23 mg/g DW), and Patate e’ moru (3.25 ± 0.40 mg/g DW). Each of these exhibited statistically significant differences from one another and from the others. Lower CGA levels were observed in Magenta love (2.05 ± 0.04 mg/g DW), Fleur bleue (1.23 ± 0.13 mg/g DW), and Blawe borges (1.18 ± 0.04 mg/g DW), which did not show significant differences among themselves.

A similar pattern was noted for chlorogenic acid derivatives, albeit at notably lower concentrations, as illustrated in Table 4. The presence of these hydroxycinnamic acids in potato peels enhances their value. Specifically, CGA is a biologically active dietary polyphenol with a range of therapeutic functions, including antibacterial, hepatoprotective, cardioprotective, anti- inflammatory, neuroprotective, anti-obesity, antiviral, and anti- hypertensive effects, along with its ability to modulate lipid and glucose metabolism [79,80,81,82,83,84,85,86,87]. Caffeic acid (CA) was found at levels approximately 100 times lower than CGA. The highest CA concentration was observed in Blue star (0.21 ± 0.01 mg/g DW), showing a significant difference from all other cultivars. Intermediate levels were recorded in Violet queen (0.15 ± 0.01 mg/g DW), Fleur bleue (0.13 ± 0.01 mg/g DW), Blawe borges (0.13 ± 0.01 mg/g DW), and Magenta love (0.12 ± 0.00 mg/g DW), which exhibited some overlap in their statistical groupings. Patate e’ moru and Rote emma had the lowest CA levels (0.031 ± 0.01 and 0.04 ± 0.00 mg/g DW, respectively), with no significant difference between them. Caffeic acid is a polyphenol commonly ingested, primarily through coffee, but also present in potato skins. Numerous studies have emphasized the beneficial effects of caffeic acid on human health, including its potential impact on cancer [88], diabetes [89], atherosclerosis [90], and neurodegenerative diseases [91], as well as its antibacterial [92] and antiviral properties [93].

Anthocyanins are the natural pigments that give potatoes their red and purple hues and have been recognized for their potential health advantages. Notably, these advantages may include reducing the risk of certain conditions, such as diabetes, cancer, cardiovascular diseases, and neurological disorders [87]. The antioxidant properties of anthocyanins are often credited with aiding in the mitigation of oxidative stress and inflammation, both of which play significant roles in the progression of various chronic diseases [94]. As indicated in Table 5, these anthocyanins consist of acylated glycosides, including pelargonidin, peonidin, petunidin, and malvidin, aligning with previous studies [37,95,96,97]. Factors such as glycosylation, acylation, polymerization degree, and interactions with other substances affect the bioavailability and potential health impacts of these anthocyanins [98,99]. The analyzed potato cultivars displayed notable qualitative and quantitative differences in their anthocyanin profiles, particularly among the petunidin derivatives. Specifically, petunidin-3-O-caffeoyl-rutinoside-5-O-glucoside was uniquely identified in the Violet queen cultivar, which had a concentration of 0.20 ± 0.00 mg/g DW. In contrast, none of the other cultivars exhibited any detectable amounts of this compound, indicating a unique biosynthetic signature for this variety.

A clearer distinction appeared with petunidin-3-O-p-coumaroyl-rutinoside-5-O-glucoside, identified in five of the seven cultivars. Violet queen emerged with a notably high concentration (7.27 ± 0.73 mg/g DW), over six times more than the next highest cultivar, Blue star (1.17 ± 0.06 mg/g DW). In contrast, Fleur bleue, Blawe borges, and Patate e’moru showed significantly lower levels, ranging from 0.11 ± 0.02 to 0.36 ± 0.05 mg/g DW. This indicates that Violet queen serves as the richest source of this anthocyanin, while the others appear to have a more limited capability for its biosynthesis. Likewise, petunidin-3-O-feruloyl-rutinoside-5-O-glucoside was detected in four cultivars, with Violet queen again exhibiting the highest level (0.28 ± 0.04 mg/g DW), followed by Blue star (0.17 ± 0.01 mg/g DW). Fleur bleue and Blawe borges had notably lower concentrations, approximately 0.05 ± 0.01 and 0.06 ± 0.00 mg/g DW, respectively. These variations underscore Violet queen and Blue star as cultivars with enhanced levels of feruloylated anthocyanins.

The pattern of malvidin-3-O-p-coumaroyl-rutinoside-5-O-glucoside distribution appeared different. The highest concentration was found in Patate e’moru (1.96 ± 0.22 mg/g DW), closely followed by Violet queen (1.19 ± 0.13 mg/g DW). The other cultivars, including Blue star, Fleur bleue, and Blawe borges, contained significantly lower amounts, with values under 170 mg/g DW. This suggests that Patate e’moru may be particularly relevant for breeding programs aimed at increasing malvidin content. Specifically, petunidin derivatives have been associated with cardiovascular health benefits, potentially aiding in blood pressure reduction and improved vascular function [100,101]. Malvidin derivatives are linked to inhibiting cancer cell growth and the prevention of specific cancer types [102,103,104,105,106].

In contrast, the anthocyanin profiles of the red potatoes Magenta love and Rote emma were notably distinct, with a prevalence of pelargonidin derivatives. Notably, pelargonidin 3-o-p-coumaroyl-rutinoside-5-o-glucoside was the most abundant anthocyanin found in Rote emma (0.99 ± 0.06 mg/g DW) and in Magenta love (0.36 ± 0.01 mg/g DW). Pelargonidin derivatives are recognized for their anti-inflammatory properties and may help mitigate neurological disorders [107,108,109]. Furthermore, Magenta love also contained peonidin-3-o-p-coumaroyl-rutinoside-5-glucoside (0.08 ± 0.00 mg/g DW). Peonidin derivatives are associated with better glycemic control and may benefit individuals with diabetes [110,111]. The anthocyanin profile corresponds with the coloration of these potato varieties, where pelargonidin and peonidin contribute to the red hue, while petunidin and malvidin are responsible for the purple tint. These specific anthocyanins may provide a broad array of health benefits. Several studies indicate that variations in phenolic profiles and antioxidant capacities among different cultivars can be affected by factors like the growth environment, genetic makeup, and fertilization methods [112,113,114].

### 3.3. PCA

**Figure 2:**
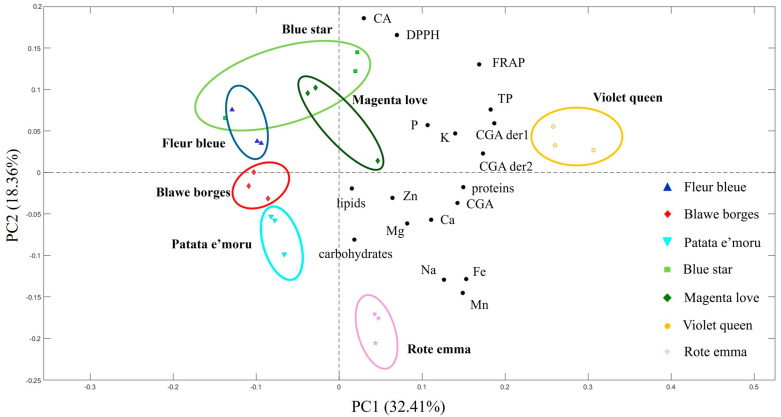
Principal component analysis (PCA) score plot of potato peel samples. PC1 explains 32.41% of the total variance, while PC2 accounts for 18.36%. Variables legend: CA (caffeic acid); CGA (chlorogenic acid); CGA der1 and CGA der2 (chlorogenic acid derivatives); TP (total polyphenols); DPPH (radical-scavenging activity (DPPH assay)); FRAP (ferric reducing antioxidant power); P (phosphorus); K (potassium); Na (sodium); Mg (magnesium); Ca (calcium); Fe (iron); Zn (zinc); Mn (manganese); lipids (total lipid content); proteins (protein content); carbohydrates (carbohydrate content).

To obtain a broader perspective and enable quicker assessments of differences among the varieties, a principal component analysis (PCA) was performed (Figure 2). The main variables considered in the PCA included phenolic compounds, antioxidant and antiradical activity, macronutrients (carbohydrates, proteins, and lipids), and minerals like Fe, K, Mg, Mn, Na, P, Zn, and Ca. Anthocyanins were excluded as they would have caused clear separation between the purple and red potato varieties. The PCA results for the analyzed potato varieties demonstrate a distinct separation of samples based on their chemical–nutritional features. The first principal component (PC1) accounts for 32.41% of the total variance, while the second principal component (PC2) accounts for 18.36%. This suggests that PC1 captures most of the variability in the dataset, while PC2 reveals additional insights into less prominent variations. The PCA biplot illustrates the distribution of various potato varieties and their links to specific chemical–nutritional parameters. Blawe borges and Patata e’moru cluster in the left quadrant of the plot, displaying a weak association with phenolic variables and antioxidant content. Rote emma is found in the lower right quadrant, indicating a stronger connection with mineral elements, such as Fe, Mn, and Na. Blue star, Magenta love, and Fleur bleue are situated in the upper-right quadrant, showing varying degrees of association with caffeic acid and antioxidant-related variables. Conversely, Violet queen is located in the far-right quadrant, signifying a unique profile with elevated levels of total phenols, chlorogenic acid derivatives, and strong antiradical activity. These findings could prove valuable for selecting varieties with specific nutritional advantages and for promoting cultivars with significant functional potential.

## 4. Conclusions

This study offers in-depth insights into the nutritional and biochemical properties of red and purple potato peels, showcasing their abundance in polyphenols, particularly chlorogenic acid (CGA) and caffeic acid, along with anthocyanins and essential nutrients. Purple potatoes stand out for their high anthocyanin levels, associated with various health benefits. Beyond their polyphenolic composition, potato peels are significant sources of macronutrients, including proteins and carbohydrates, as well as essential minerals such as potassium, calcium, and magnesium, all essential for human health. The variations in nutrient composition among different potato varieties may result from genetic and environmental factors, including soil conditions, climate, and farming practices. These findings emphasize the importance of recognizing varietal differences when assessing the nutritional value of potato peels for dietary supplements and food product enhancements. Varieties like Magenta love and Blawe borge, known for their slightly higher lipid content, could be more suitable for uses requiring a richer lipid profile, while Fleur bleue presents a lighter option. Furthermore, varieties with greater protein levels might be favored for human consumption or specific industrial purposes. Although carbohydrate content differences among the analyzed potato varieties were noted, they were not statistically significant. This study highlights the promise of red and purple potato peels as valuable agri-food by-products, encouraging sustainable nutrition practices and diminishing food waste. The diversity within the examined potato varieties opens up possibilities for developing targeted food products and high-value dietary supplements that capitalize on the health advantages of their bioactive compounds. Research like this will deepen our knowledge of the potential uses of potato peels in nutrition and the food industry, supporting sustainable growth while optimizing resource use.

## Figures and Tables

**Table 1:** Macronutrients content in colored potato tuber peels. Values are expressed as g/100 g DW (mean + SD; *n* = 3).

	Patata E’moru	Violet Queen	Fleur Bleue	Blue Star	Blawe Borges	Magenta Love	Rote Emma
Lipids	0.9 ± 0.0 ^a^	1.0 ± 0.1 ^ac^	0.7 ± 0.0 ^ad^	0.8 ± 0.1 ^acd^	1.2 ± 0.1 ^ace^	1.3 ± 0.1 ^bce^	0.8 ± 0.1 ^acd^
Proteins	24.4 ± 2.6 ^a^	31.8 ± 3.0 ^b^	13.9 ± 3.2 ^cd^	12.1 ± 0.9 ^c^	12.8 ± 0.5 ^c^	27.9 ± 2.0 ^ab^	19.5 ± 0.3 ^ad^
Carbohydrates	87.4 ± 3.0 ^a^	78.7 ± 7.9 ^a^	95.1 ± 4.2 ^a^	77.5 ± 3.0 ^a^	89.7 ± 7.4 ^a^	82.0 ± 8.0 ^a^	92.6 ± 1.2 ^a^

Means ± SD denoted by the same letter did not differ significantly at *p* ≤ 0.05, while different letters denote statistical differences with at least 95% confidence according to Tukey’s multiple comparisons test.

**Table 2:** Mineral content in colored potato tubers. Values are expressed as mg/100 g _DW_ (mean + SD; *n* = 3).

	Patata E’moru	Violet Queen	Fleur Bleue	Blue Star	Blaw Borges	Magenta Love	Rote Emma
Macro-elements							
K	1399.6 ± 22.4 ^a^	2375.4 ± 9.4 ^b^	1475.4 ± 179.6 ^a^	2135.8 ± 243.6 ^bd^	1893.4 ± 21.8 ^cde^	2130.0 ± 24.4 ^bde^	1649.1 ± 9.7 ^ae^
Ca	621.3 ± 20.2 ^a^	663.2 ± 35.7 ^a^	415.1 ± 6.4 ^bd^	488.5 ± 22.0 ^be^	370.2 ± 3.3 ^b^	602.5 ± 109.4 ^aef^	524.5 ± 11.4 ^acdf^
Mg	141.4 ± 4.5 ^a^	179.2 ± 4.5 ^b^	154.5 ± 11.7 ^a^	148.9 ± 14.0 ^a^	153.4 ± 4.0 ^a^	184.5 ± 12.7 ^b^	178.8 ± 2.8 ^b^
Na	68.6 ± 5.8 ^a^	89.9 ± 7.1 ^b^	23.5 ± 2.9 ^c^	23.6 ± 2.3 ^c^	25.6 ± 0.9 ^c^	49.0 ± 4.3 ^d^	93.0 ± 1.4 ^b^
P	14.2 ± 0.4 ^a^	20.6 ± 0.1 ^b^	14.7 ± 1.6 ^a^	33.4 ± 3.2 ^c^	13.1 ± 0.1 ^a^	20.0 ± 0.4 ^b^	19.2 ± 0.4 ^b^
Micro-elements							
Fe	18.7 ± 1.7 ^a^	51.4 ± 2.2 ^b^	12.6 ± 1.3 ^a^	21.9 ± 2.7 ^ae^	17.2 ± 1.2 ^a^	29.7 ± 1.1 ^ce^	71.3 ± 6.1 ^d^
Zn	3.3 ± 0.3 ^a^	3.7 ± 0.2 ^a^	1.6 ± 0.0 ^b^	2.8 ± 0.7 ^ab^	2.8 ± 0.2 ^ab^	3.1 ± 0.4 ^ab^	3.0 ± 0.4 ^ab^
Mn	1.3 ± 0.0 ^a^	2.1 ± 0.0 ^b^	1.2 ± 0.1 ^a^	1.3 ± 0.1 ^a^	1.2 ± 0.1 ^a^	1.8 ± 0.2 ^b^	2.6 ± 0.0 ^c^

Means ± SD denoted by the same letter did not differ significantly at *p* ≤ 0.05, while different letters denote statistical differences with at least 95% confidence according to Tukey’s multiple comparisons test.

**Table 3:** Total polyphenols content in colored potato tubers. Values are expressed as mg/g DW (mean + SD; *n* = 3). DPPH and FRAP in colored potato tubers. Values are expressed as mmol/g _DW_ (mean + SD; *n* = 3).

Cultivar	Total Polyphenols mg GA/g DW	DPPH mmol trolox/g DW	FRAP mmol FeSO4/g DW
Patate e’moru	11.64 ± 0.46 ^a^	0.044 ± 0.000 ^a^	0.090 ± 0.005 ^a^
Violet queen	27.00 ± 1.02 ^b^	0.048 ± 0.002 ^a^	0.369 ± 0.030 ^b^
Fleur bleue	8.91 ± 0.29 ^c^	0.047 ± 0.000 ^a^	0.119 ± 0.015 ^a^
Blue star	11.85 ± 0.34 ^a^	0.046 ± 0.003 ^a^	0.240 ± 0.005 ^c^
Blawe borges	6.51 ± 0.13 ^d^	0.040 ± 0.002 ^ab^	0.083 ± 0.004 ^a^
Magenta love	20.06 ± 0.37 ^e^	0.047 ± 0.000 ^a^	0.261 ± 0.030 ^dc^
Rote emma	9.48 ± 0.12 ^c^	0.039 ± 0.003 ^b^	0.095 ± 0.010 ^a^

Means ± SD denoted by the same letter did not differ significantly at *p* ≤ 0.05, while different letters denote statistical differences with at least 95% confidence according to Tukey’s multiple comparisons test.

**Table 4:** Polyphenols content at 280 nm in coulored potato tubers. Values are expressed as mg/g DW (mean + SD; *n* = 3).

λ 280 nm				
Cultivar	Chlorogenic Acid Derivative 1 ^(£)^	Chlorogenic Acid	Chlorogenic Acid Derivative 2 ^(£)^	Caffeic Acid
Patate e’moru	traces ^a^	3.25 ± 0.40 ^a^	0.24 ± 0.03 ^a^	0.031 ± 0.01 ^a^
Violet queen	0.88 ± 0.07 ^b^	10.53 ± 0.95 ^b^	2.17 ± 0.17 ^b^	0.15 ± 0.01 ^b^
Fleur bleue	0.11 ± 0.02 ^c^	1.23 ± 0.13 ^c^	0.14 ± 0.01 ^a^	0.13 ± 0.01 ^bd^
Blue star	0.53 ± 0.02 ^d^	6.45 ± 0.28 ^d^	1.29 ± 0.07 ^c^	0.21 ± 0.01 ^c^
Blawe borges	0.28 ± 0.01 ^e^	1.18 ± 0.04 ^c^	0.33 ± 0.01 ^a^	0.13 ± 0.01 ^be^
Magenta love	traces ^a^	2.05 ± 0.04 ^c^	0.30 ± 0.01 ^a^	0.12 ± 0.00 ^cde^
Rote emma	0.40 ± 0.02 ^f^	5.06 ± 0.23 ^e^	0.60 ± 0.03 ^d^	0.04 ± 0.00 ^a^

^(£)^ Chlorogenic acid derivative concentrations were expressed as Chlorogenic acid equivalent. Means ± SD denoted by the same letter did not differ significantly at *p* ≤ 0.05, while different letters denote statistical differences with at least 95% confidence according to Tukey’s multiple comparisons test.

**Table 5:** Anthocyanin content at 520 nm in coulored potato tubers. Values are expressed as mg/g DW (mean + SD; *n* = 3).

λ 520 nm						
Cultivar	Petunidin-3-O-caffeoyl-rutinoside-5-O-glucoside *	Petunidin-3-O-p-coumaryl-rutinoside-5-O-glucoside *	Petunidin-3-O-feruloyl-rutinoside-5-O-glucoside *	Malvidin 3-O-p-coumaroyl-rutinoside-5-O-glucoside *	Pelargonidin-3-O-p-coumaroyl-rutinoside-5-O-glucoside *	Peonidin-3-O-p-coumaroyl-rutinoside-5-glucoside *
Patate e’moru	-	0.11 ± 0.02 ^a^	- ^a^	1.96 ± 0.22 ^a^	-	-
Violet queen	0.20 ± 0.00	7.27 ± 0.73 ^b^	0.28 ± 0.04 ^b^	1.19 ± 0.13 ^b^	-	-
Fleur bleue	-	0.36 ± 0.05 ^a^	0.05 ± 0.01 ^ad^	0.06 ± 0.01 ^c^	-	-
Blue star	-	1.17± 0.06 ^c^	0.17 ± 0.01 ^c^	0.16 ± 0.01 ^c^	-	-
Blawe borges	-	0.32 ± 0.01 ^a^	0.06 ± 0.00 ^d^	0.03 ± 0.00 ^c^	-	-
Magenta love	-	-	-	-	0.36 ± 0.01 ^a^	0.08 ± 0.00
Rote emma	-	-	-	-	0.99 ± 0.06 ^b^	-

* Anthocyanins concentrations were expressed as malvidin 3-O-glucoside equivalent. Means ± SD denoted by the same letter did not differ significantly at *p* ≤ 0.05, while different letters denote statistical differences with at least 95% confidence according to Tukey’s multiple comparisons test.

## Data Availability

The original contributions presented in the study are included in the article, further inquiries can be directed to the corresponding author.

## References

[1] Gómez-García R., Campos D.A., Aguilar C.N., Madureira A.R., Pintado M. (2021). Valorisation of food agro-industrial by-products: From the past to the present and perspectives. J. Environ. Manag..

[2] O’Connor J., Hoang S.A., Bradney L., Dutta S., Xiong X., Tsang D.C., Ramadass K., Vinu A., Kirkham M., Bolan N.S. (2021). A review on the valorisation of food waste as a nutrient source and soil amendment. Environ. Pollut..

[3] Gebrechristos H.Y., Chen W. (2018). Utilization of potato peel as eco-friendly products: A review. Food Sci. Nutr..

[4] Djaman K., Koudahe K., Koubodana H.D., Saibou A., Essah S. (2022). Tillage Practices in Potato (*Solanum tuberosum* L.) Production: A Review. Am. J. Potato Res..

[5] Navarre D.A., Brown C.R., Sathuvalli V.R. (2019). Potato Vitamins, Minerals and Phytonutrients from a Plant Biology Perspective. Am. J. Potato Res..

[6] Joshi A., Sethi S., Arora B., Azizi A.F., Thippeswamy B. (2020). Potato Peel Composition and Utilization. Potato Nutr. Food Secur..

[7] Liang S., Mcdonald A.G. (2014). Chemical and Thermal Characterization of Potato Peel Waste and Its Fermentation Residue as Potential Resources for Biofuel and Bioproducts Production. J. Agric. Food Chem..

[8] Rai D., Aziz A., Mccue K., Rockhold D., Belknap W. (2014). Ultrasonic extraction of steroidal alkaloids from potato peel waste. Ultrason. Sonochem..

[9] Gaudino E.C., Colletti A., Grillo G., Tabasso S., Cravotto G. (2020). Emerging processing technologies for the recovery of valuable bioactive compounds from potato peels. Foods.

[10] Iriondo-Dehond M., Miguel E., Del Castillo M.D. (2018). Food byproducts as sustainable ingredients for innovative and healthy dairy foods. Nutrients.

[11] Trigo J.P., Alexandre E.M.C., Saraiva J.A., Pintado M.E. (2020). High value-added compounds from fruit and vegetable by-products–Characterization, bioactivities, and application in the development of novel food products. Crit. Rev. Food Sci. Nutr..

[12] Joly N., Souidi K., Depraetere D., Wils D., Martin P. (2021). Potato By-Products as a Source of Natural Chlorogenic Acids and Phenolic Compounds: Extraction, Characterization, and Antioxidant Capacity. Molecules.

[13] Naveed M., Hejazi V., Abbas M., Kamboh A.A., Khan G.J., Shumzaid M., Ahmad F., Babazadeh D., FangFang X., Modarresi-Ghazani F. (2018). Chlorogenic acid (CGA): A pharmacological review and call for further research. Biomed. Pharmacother..

[14] Eichhorn S., Winterhalter P. (2005). Anthocyanins from pigmented potato (*Solanum tuberosum* L.) varieties. Food Res. Int..

[15] Oertel A., Matros A., Hartmann A., Arapitsas P., Dehmer K.J., Martens S., Mock H.-P. (2017). Metabolite profiling of red and blue potatoes revealed cultivar and tissue specific patterns for anthocyanins and other polyphenols. Planta.

[16] Jansen G., Flamme W. (2006). Coloured potatoes (*Solanum tuberosum* L.)—Anthocyanin content and tuber quality. Genet. Resour. Crop Evol..

[17] Salehi B., Sharifi-Rad J., Cappellini F., Reiner Ž., Zorzan D., Imran M., Sener B., Kilic M., El-Shazly M., Fahmy N.M. (2020). The Therapeutic Potential of Anthocyanins: Current Approaches Based on Their Molecular Mechanism of Action. Front. Pharmacol..

[18] Dubois M., Gilles K.A., Hamilton J.K., Rebers P.A., Smith F. (1956). Colorimetric Method for Determination of Sugars and Related Substances. Anal. Chem..

[19] Lowry O.H., Rosebrough N.J., Farr A.L., Randall R.J. (1951). Protein measurement with the Folin phenol reagent. J. Biol. Chem..

[20] Chen Y., Vaidyanathan S. (2013). Simultaneous assay of pigments, carbohydrates, proteins and lipids in microalgae. Anal. Chim. Acta.

[21] Bligh W.J., Dyer E.G. (1959). Canadian Journal of Biochemistry and Physiology. Can. J. Biochem. Physiol..

[22] Mishra S.K., Suh W.I., Farooq W., Moon M., Shrivastav A., Park M.S., Yang J.-W. (2014). Rapid quantification of microalgal lipids in aqueous medium by a simple colorimetric method. Bioresour. Technol..

[23] D’amelia V., Sarais G., Fais G., Dessì D., Giannini V., Garramone R., Carputo D., Melito S. (2022). Biochemical Characterization and Effects of Cooking Methods on Main Phytochemicals of Red and Purple Potato Tubers, a Natural Functional Food. Foods.

[24] Singleton V.L., Rossi J.A. (1965). Colorimetry of Total Phenolics with Phosphomolybdic-Phosphotungstic Acid Reagents. Am. J. Enol. Vitic..

[25] Brand-Williams W., Cuvelier M.E., Berset C. (1995). Use of a free radical method to evaluate antioxidant activity. LWT-Food Sci. Technol..

[26] Axelrod D., Koppel D.E., Schlessinger J., Elson E., Webb W.W. (1976). Mobility measurement by analysis of fluorescence photobleaching recovery kinetics. Biophys. J..

[27] Baker M.T., Lu P., Parrella J.A., Leggette H.R. (2022). Consumer Acceptance toward Functional Foods: A Scoping Review. Int. J. Environ. Res. Public Health.

[28] Singh L., Kaur S., Aggarwal P. (2022). Techno and bio functional characterization of industrial potato waste for formulation of phytonutrients rich snack product. Food Biosci..

[29] Martinez-Fernandez J.S., Seker A., Davaritouchaee M., Gu X., Chen S. (2021). Recovering Valuable Bioactive Compounds from Potato Peels with Sequential Hydrothermal Extraction. Waste Biomass Valorization.

[30] Cozma A., Velciov A., Popescu S., Mihut C., Duma Copcea A., Lato A., Chis C., Rada M. (2024). Determination of some nutritional parameters of potato peel-preliminary research. Res. J. Agric. Sci.

[31] Sampaio S.L., Petropoulos S.A., Alexopoulos A., Heleno S.A., Santos-Buelga C., Barros L., Ferreira I.C. (2020). Potato peels as sources of functional compounds for the food industry: A review. Trends Food Sci. Technol..

[32] Kårlund A., Gómez-Gallego C., Turpeinen A.M., Palo-oja O.-M., El-Nezami H., Kolehmainen M. (2019). Protein Supplements and Their Relation with Nutrition, Microbiota Composition and Health: Is More Protein Always Better for Sportspeople?. Nutrients.

[33] Waglay A., Karboune S., Alli I. (2014). Potato protein isolates: Recovery and characterization of their properties. Food Chem..

[34] Pȩksa A., Kita A., Kulakowska K., Aniolowska M., Hamouz K., Nemś A. (2013). The quality of protein of coloured fleshed potatoes. Food Chem..

[35] Gorissen S.H.M., Crombag J.J.R., Senden J.M.G., Waterval W.A.H., Bierau J., Verdijk L.B., van Loon L.J.C. (2018). Protein content and amino acid composition of commercially available plant-based protein isolates. Amino Acids.

[36] Pinckaers P.J.M., Hendriks F.K., Hermans W.J., Goessens J.P., Senden J.M., VAN Kranenburg J.M.X., Wodzig W.K.H.W., Snijders T., VAN Loon L.J.C. (2022). Potato Protein Ingestion Increases Muscle Protein Synthesis Rates at Rest and during Recovery from Exercise in Humans. Med. Sci. Sports Exerc..

[37] Bellumori M., Silva N.A.C., Vilca L., Andrenelli L., Cecchi L., Innocenti M., Balli D., Mulinacci N. (2020). A study on the biodiversity of pigmented andean potatoes: Nutritional profile and phenolic composition. Molecules.

[38] Vaitkevičienė N. (2019). A comparative study on proximate and mineral composition of coloured potato peel and flesh. J. Sci. Food Agric..

[39] DeMartino P., Cockburn D.W. (2020). Resistant starch: Impact on the gut microbiome and health. Curr. Opin. Biotechnol..

[40] Jardon K.M., Canfora E.E., Goossens G.H., Blaak E.E. (2022). Dietary macronutrients and the gut microbiome: A precision nutrition approach to improve cardiometabolic health. Gut.

[41] Streppel M.T., Ocké M.C., Boshuizen H.C., Kok F.J., Kromhout D. (2008). Dietary fiber intake in relation to coronary heart disease and all-cause mortality over 40 y: The Zutphen Study. Am. J. Clin. Nutr..

[42] Zhang R., Han S., Zhang Z., Zhang W., Yang J., Wan Z., Qin L. (2018). Cereal Fiber Ameliorates High-Fat/Cholesterol-Diet-Induced Atherosclerosis by Modulating the NLRP3 Inflammasome Pathway in ApoE-/- Mice. J. Agric. Food Chem..

[43] Yu E.Y.W., Wesselius A., Mehrkanoon S., Brinkman M., Brandt P.v.D., White E., Weiderpass E., Le Calvez-Kelm F., Gunter M., Huybrechts I. (2020). Grain and dietary fiber intake and bladder cancer risk: A pooled analysis of prospective cohort studies. Am. J. Clin. Nutr..

[44] Cicero A.F.G., Colletti A. (2016). Role of phytochemicals in the management of metabolic syndrome. Phytomedicine.

[45] Sanjoaquin M.A., Appleby P.N., Spencer E.A., Key T.J. (2004). Nutrition and lifestyle in relation to bowel movement frequency: A cross-sectional study of 20 630 men and women in EPIC–Oxford. Public Health Nutr..

[46] Crowe F.L., Balkwill A., Cairns B.J., Appleby P.N., Green J., Reeves G.K., Key T.J., Beral V. (2014). Source of dietary fibre and diverticular disease incidence: A prospective study of UK women. Gut.

[47] Post R.E., Mainous A.G., King D.E., Simpson K.N. (2012). Dietary fiber for the treatment of type 2 diabetes mellitus: A meta-analysis. J. Am. Board Fam. Med..

[48] Jeddou K.B., Bouaziz F., Zouari-Ellouzi S., Chaari F., Ellouz-Chaabouni S., Ellouz-Ghorbel R., Nouri-Ellouz O. (2017). Improvement of texture and sensory properties of cakes by addition of potato peel powder with high level of dietary fiber and protein. Food Chem..

[49] Sharoba A., Farrag M., Abd E.A.M. (2013). Utilization of some fruits and vegetables waste as a source of dietary fiber and its effect on the cake making and its quality attributes. J. Agroaliment..

[50] Wszelaki A.L., Delwiche J.F., Walker S.D., Liggett R.E., Scheerens J.C., Kleinhenz M.D. (2005). Sensory quality and mineral and glycoalkaloid concentrations in organically and conventionally grown redskin potatoes (*Solanum tuberosum*). J. Sci. Food Agric..

[51] Weaver C.M. (2013). Potassium and health. Adv. Nutr..

[52] Jing T., Li J., He Y., Shankar A., Saxena A., Tiwari A., Maturi K.C., Solanki M.K., Singh V., Eissa M.A. (2024). Role of calcium nutrition in plant Physiology: Advances in research and insights into acidic soil conditions—A comprehensive review. Plant Physiol. Biochem..

[53] Zheng C., Yang X., Liu Z., Liu K., Huang Y. (2022). Spatial distribution of soil nutrients and evaluation of cultivated land in Xuwen county. PeerJ.

[54] Nunes J.C.S., Fontes P.C.R., Araújo E.F., Sediyama C. (2006). Potato plant growth and macronutrient uptake as affected by soil tillage and irrigation systems. Pesqui. Agropecu. Bras..

[55] Razzaque M.S. (2018). Magnesium: Are we consuming enough?. Nutrients.

[56] Andre C.M., Ghislain M., Bertin P., Oufir M., Herrera M.D.R., Hoffmann L., Hausman J.-F., Larondelle A.Y., Evers D. (2007). Andean potato cultivars (Solarium tuberosum L.) as a source of antioxidant and mineral micronutrients. J. Agric. Food Chem..

[57] Martins A.C., Oliveira-Paula G.H., Tinkov A.A., Skalny A.V., Tizabi Y., Bowman A.B., Aschner M. (2025). Role of manganese in brain health and disease: Focus on oxidative stress. Free Radic. Biol. Med..

[58] Panel E., Nda A. (2013). Scientific Opinion on Dietary Reference Values for manganese. EFSA J..

[59] Williamson G. (2017). The role of polyphenols in modern nutrition. Nutr. Bull..

[60] Li S., Wang T., Fu W., Kennett M., Cox A.D., Lee D., Vanamala J.K.P., Reddivari L. (2021). Role of Gut Microbiota in the Anti-Colitic Effects of Anthocyanin-Containing Potatoes. Mol. Nutr. Food Res..

[61] Strugała P., Urbaniak A., Kuryś P., Włoch A., Kral T., Ugorski M., Hof M., Gabrielska J. (2021). Antitumor and antioxidant activities of purple potato ethanolic extract and its interaction with liposomes, albumin and plasmid DNA. Food Funct..

[62] Charepalli V., Reddivari L., Radhakrishnan S., Vadde R., Agarwal R., Vanamala J.K.P. (2015). Anthocyanin-containing purple-fleshed potatoes suppress colon tumorigenesis via elimination of colon cancer stem cells. J. Nutr. Biochem..

[63] Ombra M.N., Fratianni F., Granese T., Cardinale F., Cozzolino A., Nazzaro F. (2015). In vitro antioxidant, antimicrobial and anti-proliferative activities of purple potato extracts (*Solanum tuberosum* cv *Vitelotte noire*) following simulated gastro-intestinal digestion. Nat. Prod. Res..

[64] Han K.H., Sekikawa M., Shimada K.-I., Hashimoto M., Hashimoto N., Noda T., Tanaka H., Fukushima M. (2006). Anthocyanin-rich purple potato flake extract has antioxidant capacity and improves antioxidant potential in rats. Br. J. Nutr..

[65] Sampaio S.L., Petropoulos S.A., Dias M.I., Pereira C., Calhelha R.C., Fernandes Â., Leme C.M., Alexopoulos A., Santos-Buelga C., Ferreira I.C. (2021). Phenolic composition and cell-based biological activities of ten coloured potato peels (*Solanum tuberosum* L.). Food Chem..

[66] Friedman M., Huang V., Quiambao Q., Noritake S., Liu J., Kwon O., Chintalapati S., Young J., Levin C.E., Tam C. (2018). Potato Peels and Their Bioactive Glycoalkaloids and Phenolic Compounds Inhibit the Growth of Pathogenic Trichomonads. J. Agric. Food Chem..

[67] Akomolafe S.F., Ajayi O.O., Agboola O.E., Adewale O.O. (2025). Comparative evaluation of the antidiabetic potential of three varieties of *Ipomoea batatas* L. Toxicol. Rep..

[68] Saenjum C., Thim-Uam A., Khonthun C., Oonlao P., Nuntaboon P., Surh Y.-J., Phromnoi K. (2025). Anthocyanins from a new hybrid sweet potato peel cultivated in Northern Thailand mitigate LPS-induced inflammation and RANKL-induced osteoporosis by regulating ROS-mediated pathways. Inflammopharmacology.

[69] de Albuquerque T.M.R., Magnani M., Lima M.D.S., Castellano L.R.C., de Souza E.L. (2021). Effects of digested flours from four different sweet potato (*Ipomoea batatas* L.) root varieties on the composition and metabolic activity of human colonic microbiota in vitro. J. Food Sci..

[70] Yin L., Chen T., Li Y., Fu S., Li L., Xu M., Niu Y. (2016). A comparative study on total anthocyanin content, composition of anthocyanidin, total phenolic content and antioxidant activity of pigmented potato peel and flesh. Food Sci. Technol. Res..

[71] Albishi T., John J.A., Al-Khalifa A.S., Shahidi F. (2013). Phenolic content and antioxidant activities of selected potato varieties and their processing by-products. J. Funct. Foods.

[72] Zhang Z., Li X., Sang S., McClements D.J., Chen L., Long J., Jiao A., Jin Z., Qiu C. (2022). Polyphenols as Plant-Based Nutraceuticals: Health Effects, Encapsulation, Nano-Delivery, and Application. Foods.

[73] Gebrechristos H.Y., Ma X., Xiao F., He Y., Zheng S., Oyungerel G., Chen W. (2020). Potato peel extracts as an antimicrobial and potential antioxidant in active edible film. Food Sci. Nutr..

[74] Mäder J., Rawel H., Kroh L.W. (2009). Composition of phenolic compounds and glycoalkaloids α-solanine and α-chaconine during commercial potato processing. J. Agric. Food Chem..

[75] Visvanathan R., Jayathilake C., Jayawardana B.C., Liyanage R. (2016). Health-beneficial properties of potato and compounds of interest. J. Sci. Food Agric..

[76] Riciputi Y., Diaz-De-Cerio E., Akyol H., Capanoglu E., Cerretani L., Caboni M.F., Verardo V. (2018). Establishment of ultrasound-assisted extraction of phenolic compounds from industrial potato by-products using response surface methodology. Food Chem..

[77] Amado I.R., Franco D., Sánchez M., Zapata C., Vázquez J.A. (2014). Optimisation of antioxidant extraction from Solanum tuberosum potato peel waste by surface response methodology. Food Chem..

[78] Javed A., Ahmad A., Tahir A., Shabbir U., Nouman M., Hameed A. (2019). Potato peel waste—Its nutraceutical, industrial and biotechnological applacations. AIMS Agric. Food.

[79] Le Y.-J., He L.-Y., Li S., Xiong C.-J., Lu C.-H., Yang X.-Y. (2022). Chlorogenic acid exerts antibacterial effects by affecting lipid metabolism and scavenging ROS in Streptococcus pyogenes. FEMS Microbiol. Lett..

[80] Cheng D., Li H., Zhou J., Wang S. (2019). Chlorogenic acid relieves lead-induced cognitive impairments and hepato-renal damage via regulating the dysbiosis of the gut microbiota in mice. Food Funct..

[81] Lukitasari M., Rohman M.S., Nugroho D.A., Widodo N., Nugrahini N.I.P. (2020). Cardiovascular protection effect of chlorogenic acid: Focus on the molecular mechanism. F1000Research.

[82] Hwang S.J., Kim Y.-W., Park Y., Lee H.-J., Kim K.-W. (2014). Anti-inflammatory effects of chlorogenic acid in lipopolysaccharide-stimulated RAW 264.7 cells. Inflamm. Res..

[83] Heitman E., Ingram D.K. (2017). Cognitive and neuroprotective effects of chlorogenic acid. Nutr. Neurosci..

[84] Kumar R., Sharma A., Iqbal M.S., Srivastava J.K. (2020). Therapeutic promises of chlorogenic acid with special emphasis on its anti-obesity property. Curr. Mol. Pharmacol..

[85] Ding Y., Cao Z., Cao L., Ding G., Wang Z., Xiao W. (2017). Antiviral activity of chlorogenic acid against influenza A (H1N1/H3N2) virus and its inhibition of neuraminidase. Sci. Rep..

[86] Suzuki A., Yamamoto N., Jokura H., Yamamoto M., Fujii A., Tokimitsu I., Saito I. (2006). Chlorogenic acid attenuates hypertension and improves endothelial function in spontaneously hypertensive rats. J. Hypertens..

[87] Lu H., Tian Z., Cui Y., Liu Z., Ma X. (2020). Chlorogenic acid: A comprehensive review of the dietary sources, processing effects, bioavailability, beneficial properties, mechanisms of action, and future directions. Compr. Rev. Food Sci. Food Saf..

[88] Pelinson L.P., Assmann C.E., Palma T.V., da Cruz I., Pillat M.M., Mânica A., Stefanello N., Weis G.C.C., de Oliveira Alves A., De Andrade C.M. (2019). Antiproliferative and apoptotic effects of caffeic acid on SK-Mel-28 human melanoma cancer cells. Mol. Biol. Rep..

[89] Oršolić N., Sirovina D., Odeh D., Gajski G., Balta V., Šver L., Jembrek M.J. (2021). Efficacy of caffeic acid on diabetes and its complications in the mouse. Molecules.

[90] Wang Y., Kaur G., Kumar M., Kushwah A.S., Kabra A., Kainth R. (2022). Caffeic acid prevents vascular oxidative stress and atherosclerosis against atherosclerogenic diet in rats. Evid.-Based Complement. Altern. Med..

[91] Zhang Y., Wu Q., Zhang L., Wang Q., Yang Z., Liu J., Feng L. (2019). Caffeic acid reduces A53T α-synuclein by activating JNK/Bcl-2-mediated autophagy in vitro and improves behaviour and protects dopaminergic neurons in a mouse model of Parkinson’s disease. Pharmacol. Res..

[92] Niu Y., Wang K., Zheng S., Wang Y., Ren Q., Li H., Ding L., Li W., Zhang L. (2020). Antibacterial effect of caffeic acid phenethyl ester on cariogenic bacteria and Streptococcus mutans biofilms. Antimicrob. Agents Chemother..

[93] Wu Z.-M., Yu Z.-J., Cui Z.-Q., Peng L.-Y., Li H.-R., Zhang C.-L., Shen H.-Q., Yi P.-F., Fu B.-D. (2017). In vitro antiviral efficacy of caffeic acid against canine distemper virus. Microb. Pathog..

[94] Panchal S.K., John O.D., Mathai M.L., Brown L. (2022). Anthocyanins in Chronic Diseases: The Power of Purple. Nutrients.

[95] Fogelman E., Oren-Shamir M., Hirschberg J., Mandolino G., Parisi B., Ovadia R., Tanami Z., Faigenboim A., Ginzberg I. (2019). Nutritional value of potato (Solanum tuberosum) in hot climates: Anthocyanins, carotenoids, and steroidal glycoalkaloids. Planta.

[96] Valiñas M.A., Lanteri M.L., Have A.T., Andreu A.B. (2017). Chlorogenic acid, anthocyanin and flavan-3-ol biosynthesis in flesh and skin of Andean potato tubers (*Solanum tuberosum* subsp. *andigena*). Food Chem..

[97] Giusti M.M., Polit M.F., Ayvaz H., Tay D., Manrique I. (2014). Characterization and quantitation of anthocyanins and other phenolics in native andean potatoes. J. Agric. Food Chem..

[98] Tierno R., López A., Riga P., Arazuri S., Jarén C., Benedicto L., Galarreta J.I.R.d. (2016). Phytochemicals determination and classification in purple and red fleshed potato tubers by analytical methods and near infrared spectroscopy. J. Sci. Food Agric..

[99] de Aguiar Cipriano P., Kim H., Fang C., Venancio V.P., Mertens-Talcott S.U., Talcott S.T. (2022). In vitro digestion, absorption and biological activities of acylated anthocyanins from purple sweet potatoes (*Ipomoea batatas*). Food Chem..

[100] Cai X.Y., Yang C., Shao L., Zhu H., Wang Y., Huang X., Wang S., Hong L. (2020). Targeting NOX 4 by petunidin improves anoxia/reoxygenation-induced myocardium injury. Eur. J. Pharmacol..

[101] Auger C., Chaabi M., Anselm E., Lobstein A., Schini-Kerth V.B. (2010). The red wine extract-induced activation of endothelial nitric oxide synthase is mediated by a great variety of polyphenolic compounds. Mol. Nutr. Food Res..

[102] Baba A.B., Nivetha R., Chattopadhyay I., Nagini S. (2017). Blueberry and malvidin inhibit cell cycle progression and induce mitochondrial-mediated apoptosis by abrogating the JAK/STAT-3 signalling pathway. Food Chem. Toxicol..

[103] Huang W.Y., Wang J., Liu Y.M., Zheng Q.S., Li C.Y. (2014). Inhibitory effect of Malvidin on TNF-α-induced inflammatory response in endothelial cells. Eur. J. Pharmacol..

[104] Shih P.H., Yeh C.T., Yen G.C. (2005). Effects of anthocyanidin on the inhibition of proliferation and induction of apoptosis in human gastric adenocarcinoma cells. Food Chem. Toxicol..

[105] Oliveira H., Wu N., Zhang Q., Wang J., Oliveira J., de Freitas V., Mateus N., He J., Fernandes I. (2016). Bioavailability studies and anticancer properties of malvidin based anthocyanins, pyranoanthocyanins and non-oxonium derivatives. Food Funct..

[106] Wang Y., Lin J., Tian J., Si X., Jiao X., Zhang W., Gong E., Li B. (2019). Blueberry Malvidin-3-galactoside Suppresses Hepatocellular Carcinoma by Regulating Apoptosis, Proliferation, and Metastasis Pathways in Vivo and in Vitro. J. Agric. Food Chem..

[107] Fu K., Chen M., Zheng H., Li C., Yang F., Niu Q. (2021). Pelargonidin ameliorates MCAO-induced cerebral ischemia/reperfusion injury in rats by the action on the Nrf2/HO-1 pathway. Transl. Neurosci..

[108] Roghani M., Niknam A., Jalali-Nadoushan M.R., Kiasalari Z., Khalili M., Baluchnejadmojarad T. (2010). Oral pelargonidin exerts dose-dependent neuroprotection in 6-hydroxydopamine rat model of hemi-parkinsonism. Brain Res. Bull..

[109] Alisavari N., Soleimani-Asl S., Zarei M., Hashemi-Firouzi N., Shahidi S. (2021). Protective effect of chronic administration of pelargonidin on neuronal apoptosis and memory process in amyloid-beta-treated rats. Avicenna J. Phytomed..

[110] Luna-Vital D.A., De Mejia E.G. (2018). Anthocyanins from purple corn activate free fatty acid-receptor 1 and glucokinase enhancing *in vitro* insulin secretion and hepatic glucose uptake. PLoS ONE.

[111] Matsui T., Ebuchi S., Kobayashi M., Fukui K., Sugita K., Terahara N., Matsumoto K. (2002). Anti-hyperglycemic effect of diacylated anthocyanin derived from Ipomoea batatas cultivar Ayamurasaki can be achieved through the alpha-glucosidase inhibitory action. J. Agric. Food Chem..

[112] André C.M., Oufir M., Hoffmann L., Hausman J.-F., Rogez H., Larondelle Y., Evers D. (2009). Influence of environment and genotype on polyphenol compounds and in vitro antioxidant capacity of native Andean potatoes (*Solanum tuberosum* L.). J. Food Compos. Anal..

[113] Hamouz K., Lachman J., Dvořák P., Jůzl M., Pivec V. (2006). The effect of site conditions, variety and fertilization on the content of polyphenols in potato tubers. Plant, Soil Environ..

[114] Ieri F., Innocenti M., Andrenelli L., Vecchio V., Mulinacci N. (2011). Rapid HPLC/DAD/MS method to determine phenolic acids, glycoalkaloids and anthocyanins in pigmented potatoes (*Solanum tuberosum* L.) and correlations with variety and geographical origin. Food Chem..

